# Development and Characterization of Calcium-Alginate Beads of Apigenin: In Vitro Antitumor, Antibacterial, and Antioxidant Activities

**DOI:** 10.3390/md19080467

**Published:** 2021-08-20

**Authors:** Mohammed F. Aldawsari, Mohammed Muqtader Ahmed, Farhat Fatima, Md. Khalid Anwer, Prakash Katakam, Abdullah Khan

**Affiliations:** 1 Department of Pharmaceutics, College of Pharmacy, Prince Sattam Bin Abdulaziz University, P.O. Box 173, Al-Kharj 11942, Saudi Arabia; moh.aldawsari@psau.edu.sa (M.F.A.); f.soherwardi@psau.edu.sa (F.F.); m.anwer@psau.edu.sa (M.K.A.); 2 Department of Pharmaceutics, Indira College of Pharmacy, Nanded 431606, Maharashtra, India; pkatakam9@gmail.com; 3 Centre of Excellence for Pharmaceutical Sciences, School of Pharmacy, KPJ Healthcare University College, Nilai 71800, Malaysia; abdullahkhan@kpjuc.edu.my

**Keywords:** apigenin, antimicrobial, beads, Ca-alginate, DSC, DPPH, FTIR, in vitro dissolution, MTT, SEM, XRD

## Abstract

The objective of this work was to develop sustained-release Ca-alginate beads of apigenin using sodium alginate, a natural polysaccharide. Six batches were prepared by applying the ionotropic gelation technique, wherein calcium chloride was used as a crosslinking agent. The beads were evaluated for particle size, drug loading, percentage yield, and in vitro drug release. Particle size was found to decrease, and drug entrapment efficiency was enhanced with an increase in the polymer concentration. The dissolution study showed sustained drug release from the apigenin-loaded alginate beads with an increase in the polymer proportion. Based on the dissolution profiles, BD6 formulation was optimized and characterized for FTIR, DSC, XRD, and SEM, results of which indicated successful development of apigenin-loaded Ca alginate beads. MTT assay demonstrated a potential anticancer effect against the breast cancer MCF-7 cell lines. The antimicrobial activity exhibited effective inhibition in the bacterial and fungal growth rate. The DPPH measurement revealed that the formulation had substantial antioxidant activity, with EC50 value slightly lowered compared to pure apigenin. A stability study demonstrated that the BD6 was stable with similar (*f*2) drug release profiles in harsh condition. In conclusion, alginate-based beads could be used for sustaining the drug release of poorly water-soluble apigenin while also improving in vitro antitumor, antimicrobial, and antioxidant activity.

## 1. Introduction

Marine-based polysaccharides originate from ocean life plants, sea animals, or marine bacteria. Marine polycarbohydrates—agar, alginic acid, chitin, chitosan, cellulose, glucan, including long-chain polymeric carbohydrates and low-molecular-weight carbohydrates—are isolated from fungi, algae, and organisms [1]. Marine-derived polymers have anticancer, antioxidant, antimicrobial, anticoagulant, and anti-inflammatory bioactivities [2,3]. A natural hydrophilic polysaccharide-alginic acid, also called algin, with metals such as sodium, calcium, and its salts, are known as alginates. Sodium alginate (sodium 3,4,5,6-tetrahydroxyoxane-2-carboxylate) is extracted from the cell wall of brown seaweed and it appears as yellowish fibrous or granular powder jelly bodies [4]. The biosynthesis of alginate is materialized by D-fructose-6-phosphate precursor, followed by epimerization of d-mannuronic residues, catalyzed by mannuronan C-5-epimerases. Sodium alginate (SAG) is viscous, has non-Newtonian consistency in water, is insoluble with ether and ethanol, and precipitates with calcium chlorides [5]. It is biodegradable and has biocompatible properties, due to which it has been widely investigated in developing drug delivery systems [6]. Nanodevices were also reported with the implication of alginate. Additionally, temporal and spatial drug release dosage forms can be developed by varying the drug-polymer proportions to achieve improved solubility, dissolution, target adherence (mucoadhesive), drug bioavailability, or bioactive compound [7]. Dry-powder/lyophilized SAG, if stored in a cool and dark place, may have a long shelf-life of several years. Cell-stabilization was achieved by forming beads, implying the entrapment process, and SAG solution was dropped into calcium chloride solution through a 0.5 mm diameter needle [8].

In the recent past, many studies were reported for the SAG in the development of drug delivery systems (DDS). formulation of SAG-based buccal mucoadhesive films of cetirizine, formulation of Amphotericin-B loaded SAG-nanospheres for effective systemic fungal infections, and formulation of diclofenac gastro retentive alginate beads for sustained release, improving the viability and stability of probiotics using SAG. Additionally, alginate-based nanomedicine, encapsulated with drugs, vaccines, and proteins, was also reported. SAG have been widely employed in the development of nanoemulsion, nanospheres, nanocrystals, dendrimers, nanoemulsion, and liposomes for efficient drug delivery at the site for better efficacy, safety, and pharmacokinetic/pharmacodynamic profiles.SAG alone and in combination with other rate-controlling polymers has been investigated for the sustained drug release, enhanced entrapment, increased dissolution rate, and bioavailability. Model chemotherapeutic agents incorporated in alginate biopolymers elicit improved penetration and deposition at the cancer site. SAGs have been widely applied for ocular and pulmonary drug delivery systems. SAGs are extensively used in the pharmaceutical and food industries as stabilizing and gelling agents [9,10,11].

Sharma et al. developed nanoparticles with stem cells to deliver 5-azacytidine in zein protein for cardiac function repairs [12]. Medical application of SAG was reported by Veerubhotla et al. in their research of a 3D printed hydrogel-based cardiovascular stent [13]. Alginate-based beads have been widely investigated for the sustained and targeted delivery of various drugs in order to enhance bioavailability [14,15,16,17]. A natural source of phytochemicals has been explored to extend the medicinal applications in the treatment of a variety of human diseases and disorders. So far, medicinal natural plant products have been considered for therapeutic rather than prophylactic use [18]. Flavonoids, as potential phytotherapeutics, are the largest phytonutrients with more than six thousand types found in almost all fruits and vegetables [19].

Apigenin (AGN) is a natural flavonoid abundantly available in tea, nuts, basil, orange, tamarind, and onion. Many studies reported the therapeutic significance of apigenin, such as antimicrobial, antioxidant, and anticancer activities, besides other multiple physiological properties [20,21].

Apigenin is a glycoside and phenolic, a flavonoid that is practically insoluble both in polar and non-polar solvents, and is generally unstable for long storage at room temperature, requiring storage at −20 °C. Moreover, flavonoids have poor absorption, being secreted by gall bladder and degraded by the colon flora [20]. Due to AGN solubility, bioavailability, and stability formulation concerns, scientists have extensively worked on it. Several formulation strategies and technologies, such as nanoparticles, nanoemulsions, nanocrystal, phytosomes, flavonosomes, ethosomes, liposomes, pharmacosomes, and cyclodextrin/phospholipids-based aggregates, have been investigated to improve the solubility, dissolution, and overall bioavailability of AGN [22,23].

Contrary to the nano-sized and lipidic drug carriers, many scientists have developed micrometer-giant particles called macro–micro range scale particles or beads. Microproducts gained popularity and wide application due to their simple manufacturing, retarded-aggregation, greater effective surface area, enhanced encapsulation, and improved solubility/dissolution-bioavailability. Additionally, beads (products) have proven to be very convenient in sustained and targeted site-specific release studies [24].

In spite of great bioactivities, one major stumbling block that has severely hampered the development of apigenin drug carriers is represented by its low solubility and poor bioavailability.

These observations have given us an impetus to develop calcium–alginate beads of apigenin using ionic gelation technique to improve antitumor, antibacterial, and antioxidant activities. Apigenin is a tactical model bioactive agent for sustained drug release dosage forms. The purpose of this study was to develop AGN-loaded calcium-alginate beads that could have the potential to improve the antitumor and antibacterial activities.

## 2. Results and Discussion

### 2.1. Particle Size Distribution

The developed Ca-alginate AGN-loaded beads ranged from 1043 ± 0.02 to 1413 ± 0.02 µm in size, and the particle size decreased with an increase in the alginate concentration. From Table 1, it is evident that particle size is inversely proportional to the concentration of alginate. The enhanced ratio of alginate in the composition of the beads forms a more rigid and compact matrix and thereby decreases the particle size. This result can be correlated with the study described by Mandal S. et al. [25].

### 2.2. Drug Content Estimation

Determination of drug content was carried out to quantify the amount of AGN in the prepared beads; it was found to be in the range of 49.87 ± 1.8 to 76.65 ± 2.0%. The results are shown in Table 1, indicating drug loading improved with an increase in the polymer proportion. Enhanced drug loading efficiency with an increase in sodium alginate concentration could be due to the greater availability of the calcium-binding sites in the anionic linear polysaccharide chain and, consequently, an additional degree of crosslinking by increasing the sodium alginate fraction [6,25].

### 2.3. Production Yield

The production yield percentage of developed Ca-alginate beads was found to be in the range of 84.98 ± 0.5% to 96.78 ± 0.9%. The yield (%) of all six batches is presented in Table 1, showing that an increase in the concentration of the alginate increases the practical yield percentage.

### 2.4. In Vitro Dissolution Study

**Figure 1:**
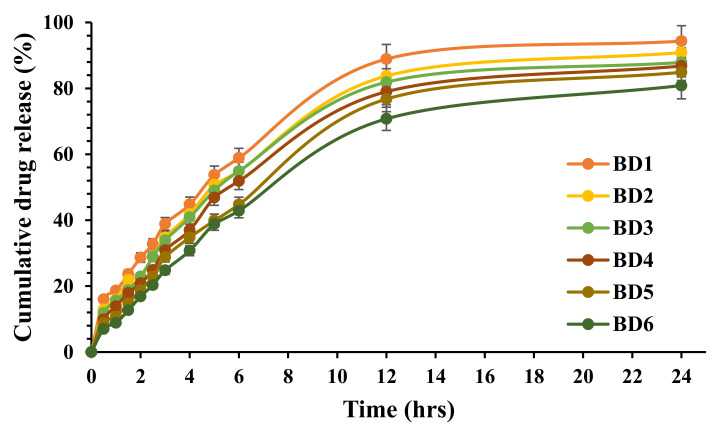
In vitro dissolution profiles of Ca-alginate-based apigenin-loaded beads.

Initial burst release effects could be due to the un-entrapped or surface-adsorbed drug and are clinically significant for the onset of action. All the formulations showed sustained drug release because of the sodium alginate crosslinked with CaCl_2_, forming the tight junction between the guluronic acid residue. The dissolution profiles for all six formulations are shown in Figure 1, and the drug release was found to be in the range of 80.87 ± 1.8% to 94.32 ± 2.3%. The distinctive drug release could be due to the variation in the SAG amount; lowering the SAG concentration led to faster drug release, and increasing the alginate concentration sustained the dissolution rate [26]. Drug release from the alginate beads also depends on the dissolution medium penetration inside the beads followed by swelling and dissolution of the alginate matrix, which resulted in the drug dissolution followed by leaching of the drug through the swollen matrix. The order of release was found to be BD1 > BD2 > BD3 > BD4 > BD5 > BD6, indicating that the drug release was retarded with an increase in the sodium alginate concentration. SAG on contact with the aqueous dissolution medium began to hydrate and swell, thereby forming the hydrogel surface layer, which regulates the influx of aqueous medium and drug dissolution. As a result of gelling, there is a decrease in water influx, leading to prolonged drug release. Enhancing the alginate concentration increased the apparent crosslinking points within the beads, producing a rigid matrix and retarding the AGN release. Apparently, increasing SAG concentration decreases the particle size due to the tight junction between CaCl_2_ and guluronic acid residues of alginate and forms a rigid compact matrix, consequently sustaining AGN release. The optimized formulation BD6 composed of 325 mg SAG and 100 mg AGN showed 80.87 ± 2.3% drug released after 24 h.

### 2.5. FTIR Spectroscopy

**Figure 2:**
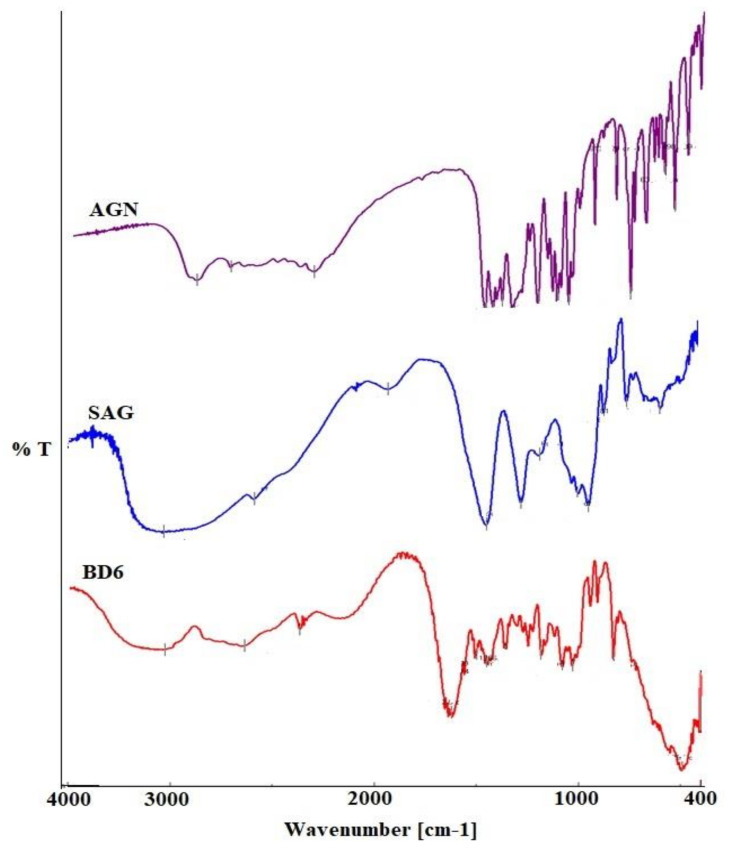
FTIR spectra of pure apigenin, sodium alginate, and optimized formulation (BD6).

FTIR (Fourier Transform Infrared Spectroscopy) analysis clarifies possible chemical interaction by the efficient identification of the components by their functional groups and bond vibrations. The IR spectra of the pure drug AGN and SAG- and AGN-loaded BDs are shown in Figure 2. The pure phytochemical apigenin spectrum exhibited characteristics peaks at 3285.14–2619.82 cm^−1^, 1349.93–2619.82 cm^−1^, 1650.77–1609.31 cm^−1^, and 1349.93–1179.26 cm^−1^, corresponding to functional groups O-H bond, C-H bending, C=O stretching, and C-C stretching, respectively.

Most of these characteristic peaks of AGN due to the functional group interaction were weakly presented in the prepared BDs, suggesting no chemical structural change of AGN in the BDs. Thus the localization of phytochemicals in the developed beads can be inferred.

### 2.6. Differential Scanning Calorimetry

**Figure 3:**
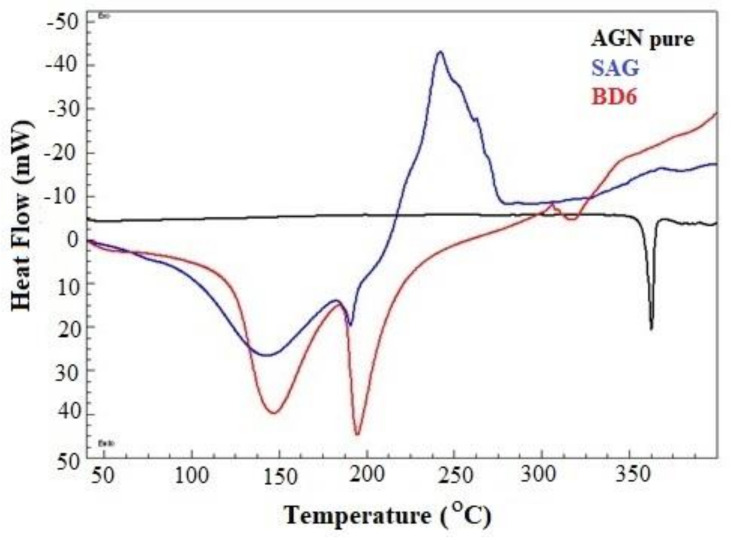
DSC thermogram of pure apigenin, sodium alginate, and optimized formulation (BD6).

Differential thermograms reflect the drug state in the BDs, endothermic peaks of AGN, and Ca-alginate BDs. The DSC spectrum of pure AGN revealed a sharp melting peak at a temperature of 362.31 °C; this endothermic peak is much closer to those reported by Alshehri et al. [27]. Sodium alginate shows two endothermic peaks at 144.13 °C and 194.95 °C. This polysaccharide also showed an exothermic broad decomposition peak at 241.53 °C. Similar peaks were reported by other investigators. The characteristic peak of SAG was not present in the optimized formulation due to the formation of beads of alginate with calcium ions, as shown in Figure 3. The differential thermogram of AGN in BD6 was diminished, suggesting the amorphous nature of the loaded drug; this disappearance of characteristic melting point also confirms negligible AGN surface adsorption [28].

### 2.7. X-ray Diffractometry

**Figure 4:**
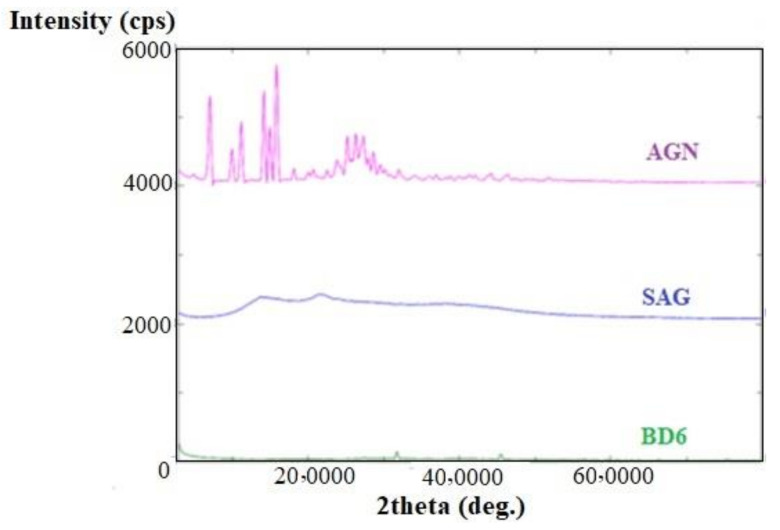
X-ray diffraction (XRD) peak of pure apigenin, sodium alginate, and optimized formulation (BD6).

XRD diffractograms of pure drug apigenin, Na-alginate alone, and apigenin-loaded BDs are shown in Figure 4. The distinct peaks of AGN in the diffractogram indicated at diffraction angles of 2θ: 7.1°, 10°, 11.2°, 14.2°, 18.2°, 23.9°, 25.7°, 26.3°, 27.4°, and 28.6°, reflecting that AGN was present in crystalline form. The SAG also exhibited some distinct peaks and broad diffuse diffraction peaks indicating their poor crystallinity. However, these crystalline diffraction peaks were not observed or diffused in XRD diffractograms of AGN-loaded microbeads, signifying that AGN would be either molecularly dispersed in polymer(s) or distributed in an amorphous form [29].

### 2.8. Scanning Electron Microscopy (SEM)

**Figure 5:**
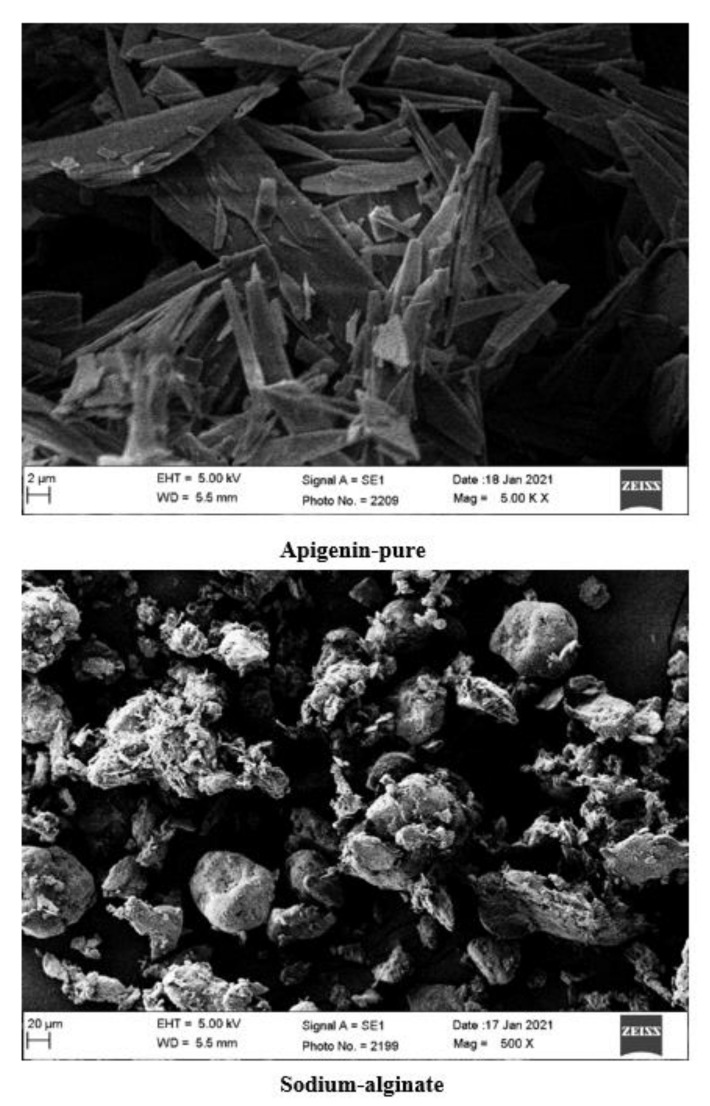

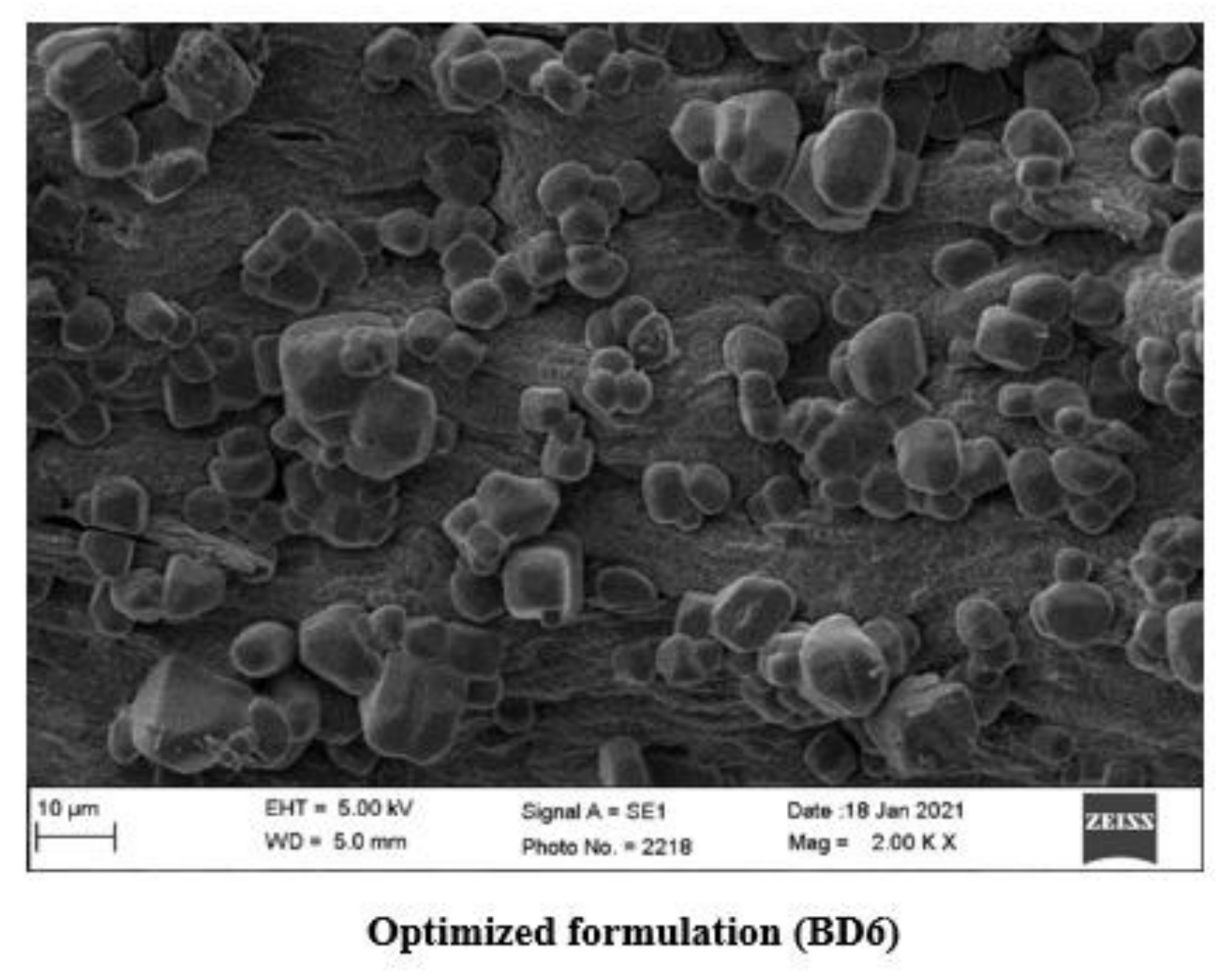
Scanning electron micrographs of pure apigenin, sodium alginate, and optimized formulation (BD6).

SEM micrographs of BDs are shown in Figure 5. The beads were found to be diffused and irregular in shape with adsorbed drug crystals over the surface. Ca-alginate-based BDs were nearly spherically elongated in shape with smooth surfaces and a hard rigid exterior.

### 2.9. In Vitro Antitumor Activity

**Figure 6:**
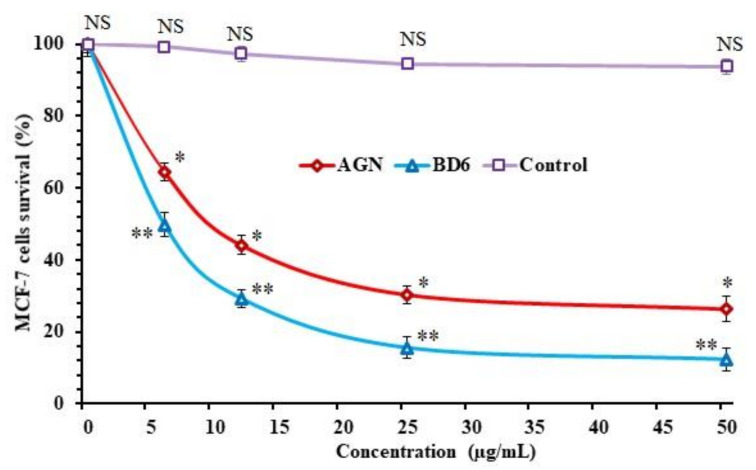
MTT assay—MCF-7 cell survival vs. the concentrations of pure apigenin and optimized formulation (BD6). Optimized formula (BD6) shows high significance (** *p* < 0.005) compared to control, significance for pure AGN vs. control (* *p* < 0.05), and non-significance between control and pure AGN (NS).

The in vitro antitumor activity of pure AGN and Ca-alginate apigenin-loaded beads (BD6) in MCF-7 cell lines was evaluated and analyzed using MTT assay. Cytotoxicity study results are shown in Figure 6. The reduction in cell survival (%) of the antitumor drug was concentration-dependent. MTT data indicate that AGN could significantly decrease the cell viability with an IC_50_ value of 9.81 µg/mL. In addition, Ca-alginate-based AGN-loaded beads also showed the highest sensitivity against the breast cancer cell (MCF-7). The IC_50_ value was 10.20 µg/mL for BD6, which was slightly higher than apigenin alone. Although AGN has a lower IC50 value to kill MCF-7 cells compared to AGN-loaded Ca-alginate beads, AGN alone may still not be suitable for clinical implication due to the solubility and bioavailability drawbacks. It was believed that AGN alone is freely available and enters the cancer cells by passive diffusion, causing apoptosis to the breast cancer cells, whereas AGN-loaded beads (BD6) increase the solubility and bioavailability, transported by internalization by active mechanism or via endocytosis. Inference of the antitumor activity represents BD6 with the enhancement of cytotoxic effects of AGN on breast cancer cells. In breast cancer cells, AGN acts as an anticancer by modulating Bcl-2 (B-cell lymphoma 2), BAX (BCL2 Associated X, Apoptosis Regulator), STAT-3 (Signal transducer and activator of transcription 3), and Akt (Ak strain transforming) protein expression [3,30].

### 2.10. Antimicrobial Activity

**Figure 7:**
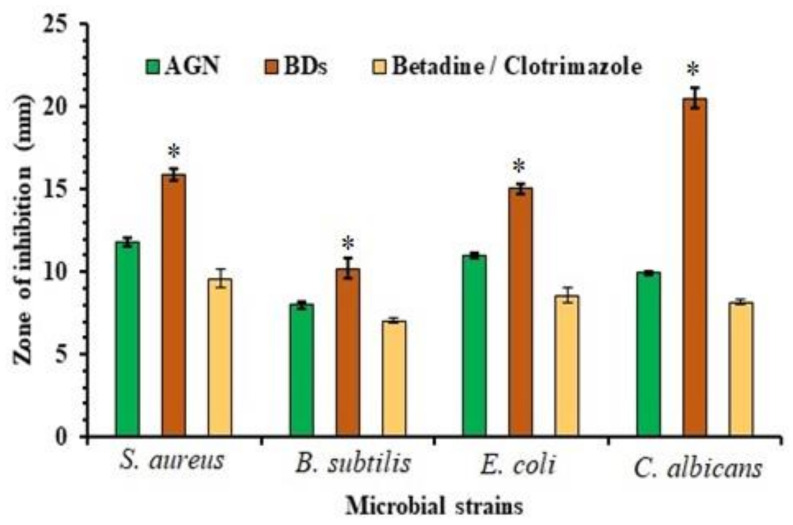
Microbial inhibitory effects of pure apigenin and optimized formulation (BD6) against the standards. The data are presented in mean ± SD (*n* = 3). Optimized formula (BD6) showed significance (* *p* < 0.05) compared to Betadine/Clotrimazole and pure AGN.

The zone of inhibition against three bacterial-one fungal strains was measured and is plotted in Figure 7. Mean diameter inhibition (mm) was found to be 11.76 ± 0.25 for *S. aureus*, 08 ± 0.20 for *B. subtilis*, and 11.03 ± 0.15 in *E. coli* for AGN, and for optimized BD6 it was 15.91 ± 0.37, 10.18 ± 0.61, and 15.02 ± 0.28 mm in *S. aureus*, *B. subtilis*, *E. coli* inoculums. However, the inhibitory effect against *C. albicans* was indicated to be 9.9 ± 0.11 and 20.51 ± 0.62 mm for AGN and BD6, respectively.

Test samples were also evaluated against the marketed products (betadine/clotrimazole) for bacterial and fungal strains. The results were found to be for 9.59 ± 0.54 mm for *S. aureus*, 7.03 ± 0.15 mm for B. subtilis, 8.56 ± 0.45 mm for E. coli and 8.13 ± 0.15 mm for *C. albicans*. Based on the antimicrobial results, it was revealed that AGN was found to be relatively more prone to *S. aureus*. Both Gram +/− were affected by AGN, which could be due to its chemical nature and cell permeability effect. The difference in its efficacy against the bacteria and fungus may be due to several possible hypotheses such as permeability barriers due to cell walls or the occurrence of enzymes in the periplasmic space of the organisms that decompose the foreign introduced molecules.

### 2.11. Antioxidant Activity

**Figure 8:**
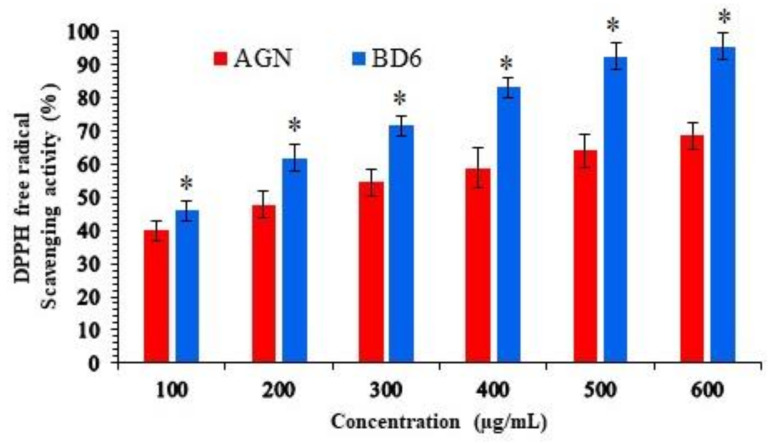
DPPH radical scavenging activity of pure AGN and BD6. The data are presented as mean ± SD (*n* = 3). Optimized formula (BD6) showed significance (* *p* < 0.05) compared to pure AGN.

The antioxidant is an important property of bioflavonoid that acts against toxic effects of free radicals, sourced from foods and biological systems that cause deleterious effects to human life. The antioxidant property was measured by estimating the free DPPH radical scavenging using spectrophotometric methods. The results of DPPH free radical scavenging (antioxidant) activity are shown in Figure 8.

The EC_50_ value of the pure apigenin was found to be 3.50 µg/mL, which was relatively higher than that of Ca-alginate optimized beads BD6 2.67 µg/mL, these observations are consistent with previously published reports [31]. Therefore, the DPPH assay indicated that the developed BD6 formulation has the potent antioxidant property as the AGN-loaded in the beads are capable of donating hydrogen (H^+^) ion to a free radical to replace odd electron (e^−^) responsible for the radical’s reactivity.

### 2.12. Stability Study

**Figure 9:**
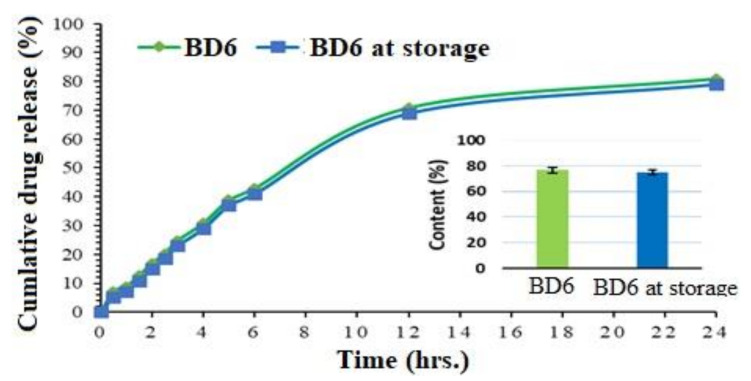
Stability dissolution study profiles and drug content estimation of the optimized formulation (BD6).

Accelerated stability indicates the quality modifications in the product due to enviornmental temperature and humidity. The results of cumulative release profiles and drug content of BD6 Ca-alginate beads exposed for stability studies (BD6 at storage) are represented in Figure 9. Before and after the stability study, drug release data fit in the similarity index, showing *f*2 value of 51.67, indicating similar drug profiles. The slightly lower drug release in the BD6 after storage condition might be due to the fact that the alginates cannot withstand higher temperatures as observed in the DSC study. Additionally, percentage drug content values were assessed for statistical significance and found with a *t*-value of 3.37 and a *p*-value of 0.03, with the result being significant at *p* < 0.05.

## 3. Materials and Methods

### 3.1. Materials

Apigenin was purchased from “Beijing Mesochem Technology Co. Pvt. Ltd. (Beijing, China)”. Sodium alginate and calcium chloride were procured from the Loba Chemie Laboratory of Reagents and Fine Chemicals, India. Roswell Park Memorial Institute medium, also known as RPMI 1640, was bought from Sigma, (St. Louis, MI, USA). All the other chemicals were of analytical grade and used as available.

### 3.2. Development of Apigenin-Loaded Calcium-Alginate Beads

Drug-polymeric solution was prepared by dissolving SAG (200–325 mg) into 50 mL of Milli-Q water, followed by dispersing the AGN (100 mg) and mixing it until smooth-viscous dispersion was achieved. A syringe fixed with a 22-gauge needle was filled with this dispersion, and the liquid was then dropped into 100 mL of calcium chloride (10% *w*/*v*) vortexed at 500 rpm. This ionotropic-gelation process was continued for 100 min, resulting in the formation of the beads (BDs), segregated by filtration and repeatedly washed to remove excess ions (CaCl_2_). BD product was then dried in an oven at 45 °C overnight and preserved in a desiccator until further analysis and characterization [25,32].

### 3.3. Particle Size Distribution

The particle size distribution (PSD) was determined by an optical microscopy technique using stage micrometer. The sample under study was immersed into liquid petrolatum and then immersed on the glass slide engraved with the scale; after placing the coverslip, the stage was adjusted and beads were visualized [33]. Mean size was calculated by using the following formula:

$$X=\frac{\sum \left(Xi\right)}{N}$$

where **X** = mean diameter of beads, **Xi** = individual diameter of beads and **N** = number of beads.

### 3.4. Drug Content Estimation

Drug content estimation was carried out by placing 10 mg of AGN-loaded beads into 100 mL of Q water. The Erlenmeyer flask was then subjected to gentle stirring at room temperature for 24 h. The solution was then centrifuged at 5000 rpm for 30 min and pre-filtered by a 0.22 μm syringe filter. The filtrate was then analyzed at λ_max_ 270 nm using spectrophotometric analysis (Jasco spectrophotometer V-630, Tokyo, Japan). The drug loading capacity of the developed beads was then calculated using the following equation [34].

$$\mathrm{Drug}\;\mathrm{loading}\;\mathrm{efficiency}\;(\%)=\frac{Total\%amount\;of\;AGN\;in\;beads}{Weight\;of\;beads\;taken}\times 100$$

### 3.5. Production Yield

Transformation of the precursors into products is a production process. Improving the yield (%) reduces the manufacturing cost and is an important factor for a profitable and sustainable business model. In this characterization parameter, AGN-loaded Ca-alginate beads collected after drying were weighed accurately, and the yield was then calculated from the below equation.

$$Yield\;\left(\%\right)=\frac{Mass\;of\;the\;Ca\;alginate\;beads\;}{Total\;weight\;of\;AGN\;and\;polymer}\times 100$$

### 3.6. In Vitro Dissolution Study

The drug release of AGN from the Ca-alginate beads was studied by placing the formulations composed of apigenin equivalent to 100 mg into the basket-type dissolution apparatus USP type-I (Erweka Dissolution DT 600H, Heusenstamm, Germany) containing 500 mL of 0.1 M HCl (pH:1.2). The shaft was rotated at 100 rpm, and the dissolution medium was maintained at 37 ± 1 °C. Five-milliliter aliquots of sample were withdrawn and replaced with the fresh medium of the same amount at predetermined time intervals. These aliquots were quantified for AGN using UV-spectroscopy at λ_max_ 270 nm. The percentage of AGN released from the beads was determined after a study of 24 h [35].

### 3.7. FTIR Spectroscopy

FTIR (Fourier Transform Infrared Spectroscopy) is a vibrational spectroscopic technique that measures the infrared absorption spectrum in the range from 4000 to 400 cm^−1^ to identify the possible chemical interaction within the drug and carrier(s) molecules. KBr pellet was prepared by compressing the triturated samples under investigation (AGN and Ca-alginate AGN-loaded beads) individually with anhydrous potassium bromide [36]. FTIR analysis of prepared samples KBr pellets were carried out into FTIR spectrophotometer (JASCO, FT/IR-4700, made in Japan).

### 3.8. Differential Scanning Calorimetry

It is a thermoanalytical technique in which AGN and optimized Ca-alginate AGN-loaded beads (BD6) weighing 2–5 mg were crimped into a hermetical aluminum pan. The sample pans were placed in a calorimeter (Scinco, N-650, Seoul, Korea) and heated from 40–400 °C with a rate of 10 °C/min under a nitrogen atmosphere (purge gas flux of N_2_ 50 mL/min) [37].

### 3.9. X-ray Diffractometry

X-ray diffraction spectrometry is a technique to evaluate the crystallographic structure of material by using diffractometer (Ultima 4-Diffractometer, Japan). The machine was equipped with high-resolution Cu-Kα radiation operating at a voltage (40 kV) and current (40 mA) at room temperature. The measurement of intensities and scattered angles of X-rays in CPCs (count per cycles) were scanned in the 2θ angle range of 0–80°. XRD difractograms were recorded for samples: pure phytoconstituent (AGN), sodium alginate, and Ca-alginate AGN-loaded beads (BD6) [38].

### 3.10. Scanning Electron Microscopy (SEM)

Micrographs of samples under study—AGN, SAG, and Ca-alginate AGN-loaded beads—were acquired by coating with a thin layer of gold with a thickness of 50 Ǻ by sputtering to increase the surface conductivity before scanning. The microstructure was observed under a scanning electron microscope (JEOL JSM-5900-LV, Tokyo, Japan) operated at 15 KV to determine the surface topology.

### 3.11. In Vitro Antitumor Activity

#### 3.11.1. Cell Culture Condition and Treatment

A human breast cancer cell line of differentiated mammary epithelium with estrogen—MCF-7—was obtained from ATCC (American Type Culture Collection), Virginia, USA. It was cultured in RPMI-1640 medium added with heat-inactivated fetal bovine serum (FBS) 10% *v*/*v*, penicillin (100 I.U/mL), and streptomycin (100 ng/mL) supplied with 90% humidified 5% CO_2_ placed inside the incubator maintained at 37 °C. The cells were seeded and cultured for 24 h in 96 well plates at a density of 2 × 10^4^ cells/well. Then, the cells were incubated with and without various concentrations of AGN alone and optimized BD6 beads [39].

#### 3.11.2. Cell Survival Assay

The antitumor activity of AGN and optimized BD6 to MCF-7 cells was measured by MTT assay using 20 μL 3-(4,5-dimethylthiazol-2-yl)-2,5-diphenyl-2H-tetrazolium bromide solution (MTT crystals dissolved in acidified isopropanol) addition in each well and vortexed. The different concentrations (6.25–50 µg/mL) of pure AGN drug, BD6, and blank beads (control) were added, and it was incubated for 24 h at 37 °C. Control wells in which no drugs were added were maintained to determine the cell survival and percentage of live cells in the cell culture.

After 24 h, the supernatant was removed, and 100 μL DMSO was added in order to dissolve the precipitate. The cell survival was estimated spectrophotometrically at an absorbance of 570 nm using an Elisa plate reader. The relative cell survival was expressed as the ratio of the absorbance of cell culture treated with pure drug AGN, group of optimized BD6 against the untreated control group. The data, in triplicate aand as mean  ±  standard deviation (SD) of survival cells and drug concentrations, were plotted to obtain the survival curve [40].

The percentage of cell survival was calculated using the following equation:

$$\mathrm{Cell}\;\mathrm{survival}\;(\%)=\frac{Optical\;Density\;\left(treated\;\right)}{Optical\;Density\;\left(control\right)}\times 100$$

Furthermore, half-maximal inhibitory concentration (IC_50_) was also estimated by using a dose–response curve, reflecting the concentration of compound required to elicit cytotoxic effect in 50% of an intact cell.

### 3.12. Antimicrobial Activity

Test organisms: Gram-positive bacteria (*Staphylococcus Aureus, Bacillus Subtilis*), Gram-negative bacteria (*Escherichia coli*), and fungal strain (*Candida albicans*) were selected for the study.

#### 3.12.1. In Vitro Antibacterial Activity

The antibacterial activity of the pure drug AGN and optimized BD6 in the selected organisms were assessed by using agar-well diffusion technique. In this method, bacterial strains were inoculated with the sterile Muller–Hinton agar and retained for 2 days at 37 °C. The samples under study were dissolved in DMSO and filled into the pre-bored wells (2 mg/well). Petri dishes were kept for 2 h at 4–7 °C (refrigerator) to enable pre-diffusion of the AGN, BD6, and Betadine Ointment. Thereafter, the plates were incubated for 24 h at 37 °C. The results were acquired by measuring the zone of inhibition and interpretations.

#### 3.12.2. In Vitro Antifungal Activity

Antifungal study was performed using 0.1 mL *C. albicans* strain spread uniformly on the sterile sabouraud dextrose agar (SSDA). The solidified SSDA was then bored at 10 mm diameter, and the samples under investigation dissolved into DMSO were filled in each well separately along with the solvent control (DMSO). The plates were kept for 1 h at 4–7 °C (refrigerator) to enable the diffusion of the samples and standard (Clotrimazole cream), followed by incubation at 27 °C for 2 days. The zone of inhibition around the well was then measured, and the results were interpreted [41].

### 3.13. Antioxidant Activity

DPPH (1,1-diphenyl-2-picryl hydrazyl) free radical scavenging activity of the optimized MBs (BD6) was tested by Blios’s technique. DPPH assay was done based on the electron transfer reaction, which produces a violet-colored solution at room temperature, which turns colorless in the presence of antioxidant moiety. Methanolic DPPH solution of 0.1 mM concentration was added to various concentrations (0.2–1 mg/mL) of samples in 1 mL:3 mL ratio. Apigenin-pure bioflavonoid (Standard), optimized Ca-alginate beads BD6 (Test) solutions and ascorbic acid (reference) solution were kept still for half an hour followed by measuring the absorbance at 517 nm [42]. The antioxidant activity was calculated by

$$\mathrm{DPPH}\;\mathrm{assay}\;(\%)\;=\;\frac{Absorbance\;of\;control\;-\;Absorbance\;of\;sample}{Absorbance\;of\;control}\times 100$$

Effective concentration (EC_50_) was also calculated by using regression analysis using log-concentration against the antioxidant activity. EC_50_ analysis was performed in Quest Graph™ EC_50_ Calculator [38].

### 3.14. Stability Study

The selected-optimized beads (BD6) were assessed for the environmental impact on the drug release by placing the 3 batches of the optimized BDs in the stability chamber (Parameter Generation and Control). The samples were exposed to room temperature (25 ± 2 °C) and accelerated stress condition (50 ± 2 °C, 75%RH) for three months and thereafter were tested for possible changes in drug content and drug release kinetics. The drug release data were then fitted to similarity index equation as suggested by SUPAC guidelines, and results were interpreted.

$${f2}_{\;=\;50\;X\;\log\text{\{}[1\;+\;(1/n)}\sum_{t-1}^{n}{\left(R_{t}-T_{t}\right)}^{2}{}_{]}{}^{-0.5}\times {}_{100\}}$$

where *f*2 means similarity index, *n* stands for dissolution time, and **R_t_** and **T_t_** denote reference (before stability study period) and test (after stability study period) dissolution values at time **t**. If the calculated *f*2 value was found to be in the range of 50–100, the sample under study was then considered to be similar in release profiles without any significant modification during the storage conditions [41].

### 3.15. Statistical Analysis

For results obtained in in vitro cell line and antimicrobial studies, statistical variations of different treatment were analyzed as per one-way analysis of variance (ANOVA, San Francisco, CA, USA) followed by post hoc Tukey’s test. *p* < 0.05 was considered as statistically significant.

## 4. Conclusions

In this investigation, Ca-alginate beads loaded with apigenin were successfully developed using the ionic gelation method. The developed beads were characterized by particle size, entrapment efficiency, process yield percentage, and dissolution study. The drug entrapment was found to increase with the increase in polymer concentration. Drug release was directly proportional to the polymer concentration and found to be sustained to an increase in the polymer concentration. The physical–chemical evaluation results revealed the apigenin beads exhibited near elongated spherical shape and could be converted into an amorphous state. The observations suggest that apigenin-loaded beads are efficient microcarriers to improve the antitumor, antibacterial, and antioxidant activities.

## Figures and Tables

**Table 1:** Composition and characterization of the ca-alginate apigenin beads.

Formulation Code	Composition			Characterization		
	AGN	SAG	CaCl_2_	PS	DL	Yield
	mg	mg	%	µm	%	%
BD1	100	200	10	1413 ± 0.02	49.87 ± 1.8	84.98 ± 0.5
BD2	100	225	10	1376 ± 0.03	55.76 ± 2.1	88.76 ± 0.6
BD3	100	250	10	1265 ± 0.01	59.87 ± 1.8	99.91 ± 0.8
BD4	100	275	10	1181 ± 0.07	66.67 ± 2.3	90.98 ± 0.3
BD5	100	300	10	1102 ± 0.05	70.34 ± 1.5	94.89 ± 0.1
BD6	100	325	10	1043 ± 0.02	76.65 ± 2.0	96.78 ± 0.9

AGN = apigenin, SAG = sodium alginate, PS = particle size, DL = drug loading.

## Data Availability

The data presented in this study are available on request from the corresponding author.
